# Global management of a common, underrated surgical task during the COVID-19 pandemic: Gallstone disease - An international survery

**DOI:** 10.1016/j.amsu.2020.07.021

**Published:** 2020-07-17

**Authors:** Tommaso Maria Manzia, Roberta Angelico, Alessandro Parente, Paolo Muiesan, Giuseppe Tisone, MEGAVID (ManagEment of GAllstone disease during coVID-19 pandemic) Clinical Investigator Group, Yousef Al Alawy, Abdul Jabba Arif, Magdy Attia, Chandra Bhati, Narendra Battula R, Glenn Kunnath Bonney, Mark Brooke-Smith, Carlos Derosas, Nicola De Liguori Carino, Stefano Ferretti, Cristina Fiorani, Dario Gherardi, Bassem Hegab, Zaki Hussain, Benedetto Ielpo, Samuele Iesari, Quirino Lai, Panagiotis Lainas, Andrea Lauterio, Alessandra Lazzaro, Ravi Marudanayagam, David Nasralla, Daniele Nicolini, Giuseppe Orlando, Damiano Patrono, Maheswaran Pitchaimuthu, Wojciech Polak, Alberto Marcacuzco Quinto, Rakesh Rai, Irene Scalera, Andrea Schlegel, Vivek Shanmugam, Alessandro Vitale, Jeannette Widmer, Deswysen Yannick

**Affiliations:** c King Salman Armed Forces Hospital, Tabuk, Saudi Arabia; d Mil Hospital Gujranwala, Gujranwala, Pakistan; e Leeds Teaching Hospital Trust, Leeds, UK; f VCU Medical Center, Richmond, VA, USA; g UF Health, FL, USA; h National University Hospital Singapore, Singapore; i Flinders Medical Centre, Adelaide, Australia; j Clinica Santa Maria, Chile + Hospital Felix Bulnes Chile, Santiago Del Chile, Chile; k Manchester Royal Infirmary, Manchester, UK; l San Camillo Forlanini Hospital, Rome, Italy; m St George's Hospital, London, UK; n Centre Hospitalier Wallonie Picarde, Tournai, Belgium; o Al Hada Armed Forces Hospital, Riyadh, Saudi Arabia; p Combined Military Hospital Kht, Pakistan; q Leon University Hospital, Leon, Spain; r Cliniques Universitaires Saint-Luc, Bruxelles, Belgium; s Sapienza University, Rome, Italy; t Antoine Beclere Hospital, AP-HP, Paris, France; u Ospedale Metropolitano Niguarda, Milan, Italy; v Colchester Hospital- ESNEFT, Colchester, UK; w Queen Elizabeth Hospital Birmingham, Birmingham, UK; x Royal Free Hospital, London, UK; y Ospedali Riuniti di Ancona, Ancona, Italy; z Wake Forest University Baptist Medical Center, Winston Salem, USA; aa AOU Città Della Salute e Della Scienza di Torino, Turin, Italy; ab Kovai Medical Center Hospital, Coimbatore, India; ac Erasmus MC University Medical Center Rotterdam, Rotterdam, Netherlands; ad University Hospital 12 de Octubre, Madrid, Spain; ae Fortis Group of Hospitals, Mumbai, India; af A.O.R.N. “A. Cardarelli”, Naples, Italy; ag University Hospital Zurich, Zurich, Switzerland; ah RPS Hospital, Chennai, India; ai Azienda Università di Padova, Padua, Italy; aj University Hospital Zurich, Switzerland; a Department of Surgical Sciences, HPB and Transplant Unit, University of Rome Tor Vergata, Rome, 00133, Italy; b Department of Hepatobiliary and Pancreatic Surgery, Queen Elizabeth Hospital, Birmingham, B15 2TH, UK

**Keywords:** Gallstone disease, Cholecystectomy, Elective surgery, COVID-19 pandemic, SARS-CoV-2

## Abstract

**Background:**

Since the Coronavirus disease-19(COVID-19) pandemic, the healthcare systems are reallocating their medical resources, with consequent narrowed access to elective surgery for benign conditions such as gallstone disease(GD). This survey represents an overview of the current policies regarding the surgical management of patients with GD during the COVID-19 pandemic.

**Methods:**

A Web-based survey was conducted among 36 Hepato-Prancreato-Biliary surgeons from 14 Countries. Through a 17-item questionnaire, participants were asked about the local management of patients with GD since the start of the COVID-19 pandemic.

**Results:**

The majority (n = 26,72.2%) of surgeons reported an alarming decrease in the cholecystectomy rate for GD since the start of the pandemic, regardless of the Country: 19(52.7%) didn't operate any GD, 7(19.4%) reduced their surgical activity by 50–75%, 10(27.8%) by 25–50%, 1(2.8%) maintained regular activity. Currently, only patients with GD complications are operated. Thirty-two (88.9%) participants expect these changes to last for at least 3 months.

In 15(41.6%) Centers, patients are currently being screened for SARS-CoV-2 infection before cholecystectomy [in 10(27.8%) Centers only in the presence of suspected infection, in 5(13.9%) routinely]. The majority of surgeons (n = 29,80.6%) have adopted a laparoscopic approach as standard surgery, 5(13.9%) perform open cholecystectomy in patients with known/suspected SARS-CoV-2 infection, and 2(5.6%) in all patients.

**Conclusion:**

In the ongoing COVID-19 emergency, the surgical treatment of GD is postponed, resulting in a huge number of untreated patients who could develop severe morbidity. Updated guidelines and dedicated pathways for patients with benign disease awaiting elective surgery are mandatory to prevent further aggravation of the overloaded healthcare systems.

## Highlights

• Since the start of the COVID-19 pandemic, elective surgeries for benign diseases have been postponed, in order to preserve healthcare resources.
• During the COVID-19 pandemic, patients with cholelithiasis, awaiting surgery, are not operated unless if complicated.
• The blockage of elective surgery will result in a large number of patients with cholelithiasis at risk of severe morbidity.
• COVID-19 free pathways for patients awaiting elective surgery are needed to prevent aggravation of healthcare systems.

## 1 Introduction

On March 11, 2020, the World Health Organization (WHO) declared the coronavirus disease-19 (COVID-19) a public health emergency with a pandemic spread [1]. As of June 20, 2020, more than 8,525,042 confirmed cases have been reported in 216 Countries across the world [1]. Almost 20% of COVID-19 patients develop severe illness, requiring hospitalization (15%) and intensive care support (5%) [2].

In many Countries, the healthcare systems are shifting the allocation of medical resources according to the evolution of the COVID-19 burden. To maintain the ability of hospitals to treat a large influx of potential COVID-19 patients, elective surgical activities addressing benign diseases have been suspended or limited [3]. However, elective surgery is not optional: indeed, being “presently non-urgent” does not mean being “unnecessary” [4]. Moreover, benign diseases scheduled for elective surgery may eventually lead to severe morbidity, which cannot always be harmlessly postponed.

Cholelithiasis is one of the most common medical issues in developed Countries, affecting roughly 20% of the population, and being symptomatic in one third of the cases [5]. Laparoscopic cholecystectomy is a well-established treatment for gallstone disease (GD) [6]. Indeed, GD is the most common gastrointestinal disorder requiring hospitalization in European Countries [6]. The annual risk of developing complications (acute pancreatitis, acute cholecystitis and cholangitis), which will cause frequent hospitalization, has been estimated to be 1–3% [5,6].

We conducted an international multi-center survey to evaluate the impact of the COVID-19 outbreak on the management of patients with GD scheduled for cholecystectomy, with the aim of defining possible strategies for their optimal management in the current pandemic scenario.

## 2 Methods

This study was conducted using an open, voluntary, Web-based qualitative survey designed in accordance with the “Consolidated Criteria for Reporting Qualitative Research” (COREQ) [7] and the “Checklist for Reporting Results of Internet E-Surveys” (CHERRIES) [8]. The survey was created by authors RA/TMM (MD, PhD) and distributed through a structured network involving an electronic mailing list through the academic account held by the University of Rome Tor Vergata, Italy. An informed consent was obtained for the participation in this study, and no incentives were offered to participants. The survey involved only healthcare professionals, and did not include any patient records or confidential data, therefore no ethical approval or patient consent form were required. The survey was dispensed from March 30, 2020 to April 5, 2020 to senior surgeons (with over 5 years of experience) working in Hepato-Prancreato-Biliary units across Europe, Asia, Oceania, and North and South America. Surgeons from Countries undergoing different pandemic phases were purposely selected. At the time of the study design, Belgium, Italy, France, the Netherlands, Spain, Switzerland and UK were on an upward trajectory of the epidemic curve, while Australia, Chile, India, Pakistan, Saudi Arabia and the US were in the early phases of local transmission and Singapore had achieved a flattening of the infection curve [1].

The 17-item online questionnaire (Table 1) was designed using Google Forms (Mountain View, CA, USA) and sent via a secure email link. The completion of the survey required approximately 10 min. All the relevant information about the questionnaire and the study was provided to the participants prior to starting, and respondents were able to withdraw from the analysis at any point. Written responses were collected and analyzed using Microsoft Excel (Redmond, WA, USA) by authors RA/TMM/AP. Participants were asked about the characteristics of their surgical units, including the type of national/local health system, the volume of GDs treated at the Center, the rate of GD symptomatic patients versus those presenting complications, the waiting list times, and the elective surgical approaches to cholecystectomy. Participants were asked whether they had changed their usual management of GD patients after the WHO declaration of a viral pandemic (March 11, 2020), and which strategy they had eventually adopted based on the local context. GD was defined as the presence of gallstones causing biliary colic, and complicated GD as gallstones causing complications including cholecystitis, acute pancreatitis, bile duct obstruction and cholangitis.

**Table 1:** Questionnaire used for the survey on the management of gallstone disease during the COVID-19 pandemic.

Question	Multiple choice answer
1. In your Country, which kind of health system do you have?	Public health system, with universal coverage
	Mainly public health system, but without universal coverage
	Mainly private health system
	Only private health system
2. Which type of hospital do you work for?	Public University Hospital
	Private University Hospital
	Public Hospital
	Private Hospital
	Other^a^
3. How many patients with gallstone disease are referred to your unit in one month?	<10
	10–20
	>20
4. How many patients with gallstone diseases are frequently symptomatic?	>25%
	25–50%
	>50%
5. How many patients with gallstone disease are currently on the waiting list for cholecystectomy in your Center?	<50
	50–100
	>100
6. How many patients with gallstone disease on your waiting list for cholecystectomy have had complications (acute pancreatitis, gallbladder empyema, common bile duct obstruction, cholangitis, etc.)?	<25%
	25–50%
	50–75%
	>75%
7. How many patients with gallstone disease on your waiting list for cholecystectomy have been previously treated by ERCP for acute pancreatitis?	<25%
	25–50%
	50–75%
	>75%
8. How long is the average waiting time for cholecystectomy in your unit?	<15 days
	15–30 days
	>30 days
	Other^a^
9. How many cholecystectomies are performed in your unit during one month (before the COVID-19 pandemic)?	<10
	10–20
	>20
10. How many cholecystectomies are performed in ambulatory regimen (one day admission) before the COVID-19 pandemic?	<25%
	25–50%
	50–75%
	>75%
11. Since COVID-19 pandemic, how did your hospital change its organization?	My hospital is exclusively dedicated to COVID-19 patients
	My hospital has restricted areas dedicated to COVID-19 patients
	My hospital doesn't treat COVID-19 patients
12. Since the COVID-19 pandemic, did you change the management of patients with gallstone disease waiting for cholecystectomy?	No
	Yes, we are not currently operating patients with gallstone disease
	Yes, we currently operate only patients with complicated gallstone disease
	Yes, the waiting list for cholecystectomy has been transferred to other hospitals
13. If you changed the management of patients with gallstone disease waiting for cholecystectomy, for how long do you expect that the change will last?	1 month
	2–3 months
	3–6 months
	>6 months
	Other^a^
14. Do you routinely screen for SARS-CoV-2 infection patients with gallstone disease before cholecystectomy?	No
	Yes, all patients
	Yes, only patients with respiratory symptoms or suspect of SARS-CoV-2 infection
	Other^a^
15. Since the COVID-19 pandemic, what is the decrease in the percentage of cholecystectomy for patients with gallstone disease in your unit?	0%
	<25%
	25–50%
	50–75%
	75–100%
16. Since the COVID-19 pandemic, did you change the surgical technique for cholecystectomy from laparoscopy to open procedure?	No
	Yes, I'm currently doing open cholecystectomy in all patients
	Yes, I'm currently doing open cholecystectomy only in patients with known or suspected SARS-CoV-2 infection
17. How would you face the management of patients with gallstone disease waiting for cholecystectomy during the COVID-19 pandemic?	I suggest to postpone cholecystectomy for patients with gallstone disease until the end of COVID-19 outbreak in our Country
	I suggest to maintain the service of cholecystectomy only for patients with complicated gallstone disease, ensuring dedicated pathway and operating theatres
	I suggest to maintain the service of cholecystectomy for all patients with gallstone disease, ensuring dedicated pathway and operating theatres
	I suggest to move all patients with gallstone disease waiting for cholecystectomy in Hospital not treating COVID-19 patients
	Other^a^

**Abbreviations:** COVID-19, coronavirus disease-19; ERCP, endoscopic retrograde cholangiopancreatography.

a = in this filed all participants had the opportunity to make comments.

## 3 Results

Out of the 40 surgeons invited to participate in the survey, 36 responses from 14 Countries were obtained (response rate 90%), all of which were included in the analysis. The majority (n = 32, 88.8%) of surgeons were based in Countries with a public health system, with 24 (66.7%) of them working in university hospitals, 7 (19.4%) in public hospitals, 3 (8.3%) in private hospitals and 2 (5.6%) in military hospitals.

Twenty-three (63.9%) participants worked in high-volume units (>20 with referred GD patients/month), 8 (22.2%) in medium-volume units (10–20 patients/month) and 5 (13.9%) in low-volume units (<10 patients/month). Twenty-seven (75%) surgeons reported that the GD patients listed for cholecystectomy in their Centers suffered from frequent symptoms, and 10 (27.8%) stated that more than 25% of their patients had experienced complications; less than 25% of these patients had already undergone endoscopic retrograde cholangiopancreatography for acute pancreatitis. Before the COVID-19 pandemic, the average number of cholecystectomies performed in one month was >20 in 18 (50%) Centers, 10–20 in 12 (33.3%) Centers, and <10 in 6 (16.7%) Centers. In half of the units examined (n = 19, 52.8%), the average waiting time for patients scheduled for elective cholecystectomy was >1 month, and only 14 (38.8%) Centers made a significant use (>50%) of the ambulatory regimen for cholecystectomy (Table 2).

**Table 2:** Overview of the activities in patients with gallstone disease at the Centers surveyed before the COVID-19 pandemic.

Country (number of Centers)*	Type of health system	Number of patients with GD referred in one month	Number of patients with GD on the waiting list for cholecystectomy	Average time on the waiting list for cholecystectomy (days)	Number of cholecystectomies performed in one month	Number of cholecystectomies performed in ambulatory regimen
**AUSTRALIA (1)**	Public health system with universal coverage	>20	<50	>30	>20	<25%
**BELGIUM (3)**	Mainly public health system, but without universal coverage	10-20 (2) >20 (1)	<50 (3)	<15 (1) 15-30 (1) NA (1)	10-20 (1) >20 (2)	<25% (2) 25–50% (1)
**CHILE (1)**	Mainly public health system, but without universal coverage	>20	>100	15–30	>20	>75%
**FRANCE (1)**	Public health system with universal coverage	>20	<50	15–30	10–20	>75%
**INDIA (3)**	Mainly private health system	10-20 (1) >20 (2)	<50 (3)	<15 (3)	10-20 (1) >20 (2)	<25%
**ITALY (8)**	Public health system with universal coverage	<10 (3) 10-20 (4) >20 (1)	<50 (7) >100 (1)	15-30 (3) >30 (5)	<10 (4) 10-20 (4)	<25% (6) 25–50% (1) 75% (1)
**NETHERLANDS (1)**	Public health system with universal coverage	<10	<50	>30	10–20	>75%
**PAKISTAN (2)**	Mainly public health system, but without universal coverage	>20	<50	15–30	>20	<25% (1) >75% (1)
**SAUDI ARABIA (2)**	Public health system with universal coverage	>20	<50	15-30 (1) >30 (1)	>20	<25% (1) 25–50% (1)
**SINGAPORE (1)**	Public health system with universal coverage	>20	<50	15–30	>20	25–50%
**SPAIN (2)**	Public health system with universal coverage	>20	50-100 (1) >100 (1)	>30	10-20 (1) >20 (1)	<25%
**SWITZERLAND (2)**	Public health system with universal coverage	>20	<50 (1) 50-100 (1)	<15 (1) >30 (1)	10-20 (1) >20 (1)	<25% (1) 25–50% (1)
**UK (6)**	Public health system with universal coverage	>20 (6)	50-100 (2) >100 (4)	>30 (6)	<10 (1) 10-20 (1) >20 (4)	50–75% (2) >75% (4)
**USA (3)**	Mainly private health system	<10 (1) 10-20 (1) >20 (1)	<50 (2) 50-100 (1)	<15 (2) >30 (1)	<10 (1) 10-20 (1) >20 (2)	<25% (1) 50–75% (1) >75% (1)

**Abbreviations:** GD, gallstone disease. *Number of Centers that participated in the survey for each Country. () = In brackets the number of responders for each Country.

Since the declaration of the COVID-19 pandemic, 32 (88.9%) participants stated that their hospital had dedicated areas restricted to Severe Acute Respiratory Syndrome Coronavirus 2 (SARS-CoV-2)-infected patients. Two (5.6%) hospitals became exclusively dedicated to COVID-19 patients, while 2 (5.6%) are not treating COVID-19 patients as yet.

Notably, the majority (n = 26, 72.2%) of surgeons reported an alarming decrease in the cholecystectomy rate for GD since the start of the pandemic, regardless of the Country: 19 (52.7%) participants affirmed that they were not currently performing any cholecystectomy, 7 (19.4%) reported a 50–75% decrease in their surgical activity, and 10 (27.8%) reduced their rate of cholecystectomy by 25–50%. Only one (2.8%) participant stated that his/her GD surgical activity was not reduced (Table 3).

**Table 3:** Results of the survey on the change in the surgical activities for gallstone disease during the COVID-19 pandemic.

**Country (number of Centers)**^a^	**Since COVID-19, how did you change the management of patients with GD?**	**Since COVID-19, what is the decrease in the rate of cholecystectomy for GD in your unit?**	**Do you routinely screen patients for SARS-CoV-2 before cholecystectomy?**	**Did you change the surgical technique of cholecystectomy from laparoscopic to open?**	**Expected duration of the modified approach**
**AUSTRALIA (1)**	-Surgery only for complicated GD	<25%	- No	- No	>6 months
**BELGIUM (3)**	-Surgery only for complicated GD	50–75% (2) 75–100% (1)	- Yes, all pts (1) - Yes, only in suspected pts§ (1) - No (1)	- No	2–3 months (2) 1 months (1)
**CHILE (1)**	-Surgery only for complicated GD	75–100%	- No	- No	3–6 months
**FRANCE (1)**	-Surgery only for complicated GD	75–100%	- No	- No	2–3 months (1)
**INDIA (3)**	-Surgery only for complicated GD (2) -No surgery for GD (1)^b^	<25% (1) 50–75% (1) 75–100% (1)	- No (2) - Yes, only in suspected pts (1)	- No (2) - Yes, open chole only in pts with known or suspected SARS-CoV-2 infection (1)	1 months (2) 2–3 months (1)
**ITALY (8)**	-Surgery only for complicated GD (4) -No surgery for GD (4)^b^	25–50% (1) 50–75% (1) 75–100% (6)	- No (4) - Yes, all pts (2) - Yes, only in suspected pts (2)	- No (7) - Yes, open chole only in pts with known or suspected SARS-CoV-2 infection (1)	2–3 months (2) 3–6 months (6)
**NETHERLANDS (1)**	-Surgery only for complicated GD	75–100%	- Yes, all pts	- No	Not predictable
**PAKISTAN (2)**	-Surgery only for complicated GD	50–75% (1) 75–100% (1)	- No (1) - Yes, only in suspected pts (1)	- Yes, open chole only in pts with known or suspected SARS-CoV-2 infection (1) - Yes, open chole in all patients (1)	2–3 months
**SAUDI ARABIA (2)**	-Surgery only for complicated GD	<25% (1) 50–75% (1)	- No (1) - Yes, only in suspected pts (1)	- No	2–3 months
**SINGAPORE (1)**	-Surgery only for complicated GD	<25%	- Yes, only in suspected pts	- No	3–6 months
**SPAIN (2)**	-Surgery only for complicated GD (1) -No surgery for GD (1)^b^	<25% (1) 75–100% (1)	- No	- No (1) - Yes, open chole only in pts with known or suspected SARS-CoV-2 infection (1)	2–3 months
**SWITZERLAND (2)**	-Surgery only for complicated GD (1) -No surgery for GD (1)^b^	<25% (1) 50–75% (1)	- No	- No	2–3 months
**UK (6)**	-No surgery for GD (6)^b^	75–100%	- No (3) - Yes, only in suspected pts (2) - Yes, all pts (1)	- No (4) - Yes, open chole only in pts with known or suspected SARS-CoV-2 infection (1) - Yes, open chole in all patients (1)	2–3 months (2) 3–6 months (2) >6 months (2)
**USA (3)**	-Surgery only for complicated GD	<25% (2) 75–100% (1)	- Yes, only in suspected pts (1) - No (2)	- No	1 month (1) 2–3 months (2)
**OVERALL (36)**	-Surgery only for complicated GD (23) -No surgery for GD (13)^b^	0% (1) <25% (8) 25–50% (1) 50–75% (7) 75–100% (19)	- No (21) - Yes, only in suspected pts (10) - Yes, all pts (5)	- No (29) - Yes, open chole only in pts with known or suspected SARS-CoV-2 infection (5) - Yes, open chole in all patients (2)	1 month (4) 2–3 months (18) 3–6 months (10) >6 months (3) Not predictable (1)

**Abbreviations:** chole, cholecystectomy; GD, gallstone disease; SARS-CoV-2, severe acute respiratory syndrome coronavirus 2.

§“Suspected patients” include patients with respiratory symptoms or suspected of SARS-CoV-2 infection. () = In brackets the number of responders for each Country.

a Number of Centers that participated in the survey for each Country.

b No surgery for GD = either for complicated or not complicated GD.

None of the participants reported that their unit currently operated on patients with GD, unless complications had occurred. In Countries with a higher SARS-CoV-2 spread at the time of the survey – such as Italy and the UK [1] – participants stated that not even patients with complicated GD were currently being considered for surgery. The large majority of surgeons (n = 32, 88.9%) said that they expected these changes to last for at least 3 more months. When asked how they would manage GD patients waiting for cholecystectomy during the COVID-19 outbreak, 21 (58.3%) participants said that they would maintain the service only for patients with complicated GD, with dedicated pathways and operating rooms, 10 (27.8%) declared that they would recommend postponing all interventions until the end of the outbreak, and 5 (13.9%) reported that they would transfer GD patients to a COVID-19-free hospital.

The survey showed that the proportion of patients scheduled for GD surgery undergoing a screening for the SARS-CoV-2 infection varies depending on the COVID-19 burden in each Country; patients are currently being screened for infection in 15 (41.6%) Centers [all from Countries with a high incidence of infection transmission (1)] before surgery, in 10 (27.8%) only in the presence of suspected infection, and in 5 (13.9%) routinely.

As for the cholecystectomy technique, the majority of surgeons (n = 29, 80.6%) have currently adopted a laparoscopic approach as standard surgery, 5 (13.9%) participants use an open approach in patients with known or suspected SARS-CoV-2 infection, and 2 (5.6%) perform an open cholecystectomy in all patients.

## 4 Discussion

Since the COVID-19 outbreak reached pandemic levels, recommendations for the fair allocation of resources should be based on the following principles: maximizing the benefits of medical interventions, protecting and preserving the healthcare workforce, paying attention not to allocate resources only on a first-come/first-served basis, being responsive to evidence, and applying the same principles to both COVID-19 and non-COVID-19 patients [9].

International surgical societies recommend to avoid elective surgical procedures, including cholecystectomy, in order to ration the use of the medical resources (ICU beds, ventilators, and personal protective equipment) and the healthcare professionals needed to face the COVID-19 pandemic [3]. Conversely, emergency and oncological surgeries should be continued [3].

Our survey depicts a wide adherence to this rule. Cholecystectomy is, in fact, reserved only for patients with acute GD-related complications. Of these, 70% of cases are treated surgically, while 30% are managed by medical treatment alone. Although these results could be expected, surprisingly, in terms of cancellation of GD surgery schedules, responses did not differ among participants from Countries at different stages of COVID-19 spread. This results in a large number of patients affected by uncomplicated but symptomatic gallstones – 50% of whom suffering from frequent colic – and by the uncertainty about when definitive surgery could be performed. This scenario may conceivably increase the risk of developing gallstone-related complications, such as acute cholecystitis, cholangitis and acute pancreatitis, which would inevitably require hospitalization, and eventually urgent care [5,6]. Gallstone-related acute pancreatitis – which has an annual incidence ranging from 15 to 40/100,000 across different Countries [10,11] – could potentially be a life-threatening complication, requiring urgent endoscopic or surgical intervention. Additionally, a more advanced disease at the time of surgery may result in increasingly morbid operations, which are associated with prolonged hospital stay and higher costs [4]. Since the global healthcare community has never faced a dramatic scenario such as the COVID-19 pandemic, it is difficult to forecast the effects of untreated GD and the development of its related complications during and after COVID-19. Recent US reports [12,13] show that the visits to the Emergency Department declined up to 50% during the early period of the COVID-19 pandemic, due to the patients’ fear of contracting the SARS-CoV-2 infection. This led to an increase in the mortality and morbidity rates for life-treating diseases, such as cardiovascular events [14]. However, so far there are no specific data available on GD-related complications [12]. Based on pre-pandemic data, assuming that the current crisis could last for 10 weeks [15], over 23,400 cholecystectomies in Italy and 18,200 in UK would be delayed, since almost all GD surgeries in these Countries have been suspended [16].

This huge number of untreated GDs suggests that, in the current COVID-19 pandemic, the surgeons’ responsibility should not only be to care for emergency or oncological cases, but also to prevent patients with benign, uncomplicated diseases from developing complications, further aggravating the pressure on the health systems.

The surgical management of GD patients should be carefully planned, considering: 1) the expectation of SARS-CoV-2 spread in each Country; 2) the organization of the hospitals and resources available during the COVID-19 pandemic; 3) the clinical status of patients on the surgical waiting list, who should be monitored through regular phone consultations and telemedicine.

Since this survey shows that 90% of hospitals are treating COVID-19 patients, dedicated COVID-19-free pathways and precautions should be preserved for all other cases [17]. Most surgeons agree on the need to proceed with surgery only in GD patients with complications. For uncomplicated symptomatic patients, who are treatable by laparoscopic cholecystectomy – which usually doesn't require a post-operative ICU stay – a possible alternative might be the wider use of ambulatory surgery. The current survey shows that only 39% of Centers routinely perform cholecystectomy in a day case ambulatory setting. It is well-known that an ambulatory regimen is both feasible and cost-effective [18,19], therefore this strategy could be a good option in the current pandemic to limit any further hospital overload. However, the high turnover of patients and healthcare providers in ambulatory care services, despite reducing hospitalization, could be a further potential source of viral infection among the medical staff in the course of their daily activity. In this regard, it might be safer to ask patients to self-quarantine for 14 days prior to surgery, as well as to obtain a PCR negative test before the operation. This, combined with a wider use of personal protective equipment (PPE) and COVID-19-free dedicated surgical pathways, could improve the number of ambulatory admissions.

Alternatively, according to the initial experience of China and Italy [17], in hospitals lacking day case surgical services, patients with GD awaiting elective cholecystectomy should be transferred to other COVID-19-free hospitals.

Since the start of the COVID-19 pandemic, concerns have been raised regarding the safety of smoke evacuation during laparoscopic surgery, as the virus could potentially spread during laparoscopy [17]. The results of our survey do not show any dramatic change in the GD surgical technique during the pandemic. In fact, only 5.6% of participants perform an open cholecystectomy in all patients, whereas 80.6% use a standard laparoscopic approach. However, 13.9% of participants would use an open approach in patients with known or suspected SARS-CoV-2 infection. In this regard, The Royal College of Surgeons recommends to consider laparoscopy only in selected individual cases, where the clinical benefit to the patient substantially exceeds the risk of potential viral transmission to the surgeons and the theatre teams in that particular situation [20]. However, open cholecystectomy might lead to an increased risk of post-operative infection, and therefore a prolonged hospitalization, whereas laparoscopic cholecystectomy can be safely performed as a day case surgical procedure, thus reducing the hospitalization time [5,6]. Recently, even in confirmed or suspected COVID-19 cases, some authors suggest to use safe and reliable filtering and evacuation systems for pneumoperitoneum gases, which could decrease the chances of viral spreading, enabling the safe use of the laparoscopic approach [21]. In our opinion, protecting the healthcare teams with a safety-first approach is mandatory; thus, with the PPE and adequate smoke evacuation systems (filters, careful deflating, traps) [21], laparoscopic cholecystectomy for GD could be safely performed. This would lead to a reduced hospitalization time, which is extremely helpful for some categories of patients affected by GD (i.e. high BMI and the elderly). In addition, it could potentially spare medical personnel and resources. However, the circulation of information on the COVID-19 transmission is continuously evolving, and it is likely that further data will come out in the next future to provide evidence-based recommendations for the surgical practice.

The results of this survey are limited by the small number of participants for each Country, the heterogeneity of the surgical Centers and the rapidly changing scenario of the COVID-19 pandemic. Furthermore, since the survey was conducted during the initial four weeks of the pandemic, it lacks the data regarding the outcome of GD complications and their treatment *after* the COVID-19 outbreak. To address this issue, further studies are needed.

To the best of our knowledge, this is the first study to show the potential impact of the COVID-19 pandemic on a benign disease requiring elective surgery, such as symptomatic GD. In the ongoing COVID-19 emergency, there is a substantial risk that many other benign diseases – which may develop complications if left without surgical treatment until the end of the pandemic – could have unexpected consequences for the healthcare systems. Therefore, as for life-saving procedures, it is of paramount importance to generate updated guidelines for the fair management of patients with benign disease in need of elective surgery during the pandemic, or immediately thereafter.

## Provenance and peer review

Not commissioned, externally peer reviewed.

## Data statement

The authors confirm that the data supporting the findings of this study are available within the article. Further details of this study are available on request from the corresponding author.

## Funding

None of the authors received any funding, direct or indirect, related to the achievement of this work from the following organizations: National Institutes of Health (NIH); Wellcome Trust; Howard Hughes Medical Institute (HHMI); and other(s). The authors would like to thank Novartis Farma Italia for their editorial assistance.

## Ethical approval

As the study is a qualitative survey among health care professionals without any involvement on patient data, no approval was required.

## Consent

No patients were involved in the study. An informed consent was obtained for the participation in this study from the surgeons survived.

## Author contribution

Tommaso Maria Manzia: Conceptualization, Methodology, Writing-Original draft preparation, Formal analysis.

Roberta Angelico: Conceptualization, Methodology, Writing-Original draft preparation, Formal analysis.

Alessandro Parente: Data curation, Formal analysis, Visualization.

Paolo Muiesan: Conceptualization, Writing-reviewing and Editing.

Giuseppe Tisone: Conceptualization, Supervision, Writing-reviewing and Editing.

MEGAVID (ManagEment of GAllstone disease during coVID-19 pandemic) Clinical Investigator Group: Resources, Writing-reviewing and Editing.

## Registration of research studies

1. Name of the registry: Research Registry
2. Unique Identifying number or registration ID: researchregistry5549
3. Hyperlink to your specific registration (must be publicly accessible and will be checked):

https://www.researchregistry.com/browse-the-registry#home/registrationdetails/5ea81b67c7420a0015c20023/

## Guarantor

Roberta Angelico.

Tommaso Maria Manzia.

## Declaration of competing interest

All the authors declare that there are no conflicts of interest regarding the publication of this manuscript.
